# New taxa and revisionary systematics of alcyonacean octocorals from the Pacific coast of North America (Cnidaria, Anthozoa)

**DOI:** 10.3897/zookeys.283.4803

**Published:** 2013-04-03

**Authors:** Gary C. Williams

**Affiliations:** 1 Department of Invertebrate Zoology and Geology, California Academy of Sciences, 55 Music Concourse Drive, San Francisco, California, 94118, USA

**Keywords:** *Alcyonium*, *Cryptophyton*, *Gersemia*, Octocorallia, northeastern Pacific, plexaurid gorgonian, soft corals, taxonomy

## Abstract

A taxonomic assessment of four species of octocorals from the northeastern Pacific Ocean (British Columbia to California) is provided. Included here are a new species of clavulariid stolonifieran *Cryptophyton*, a new species of the nephtheid soft coral *Gersemia*, an undetermined species of soft coral in the genus *Alcyonium* that has been referred in the literature by several other names, and a new genus is named for a plexaurid sea fan originally described in the Indo-Pacific genus *Euplexaura*. Discussions are included that compare the species to related taxa, or provide revisionary assessments.

## Introduction

Bayer (1981a: 7–9) reviewed the present status of knowledge of octocorals in the major geographical regions of the world and established four categories representing broad levels of taxonomic knowledge – essentially complete, moderately well-known, poorly known, and minimally known. He regarded the geographic region of the western coast of the Americas south to the Gulf of Panama as, “Moderately well-known: where there is extensive literature, but many more species remain to be described and taxonomic problems to be solved, and the major patterns of distribution must yet be worked out. Much descriptive work remains to be done before ecological and experimental studies can proceed at an effective level.” In spite of this, he lists a paucity of works in the literature – only five articles that treat the fauna, three of which cover tropical Central America (from the Gulf of California to Panama), while only two treat California – Nutting (1909) and Kükenthal (1913). The coasts of Oregon, Washington, and British Columbia are for the most part absent from the taxonomic literature. The calcaxonian octocorals of the eastern Pacific were treated by Cairns (2007) and the book chapter of Williams (2007) dealing with shallow water octocorals of the region, were added subsequently. These four works taken together, amount to the most inclusive taxonomic treatment to date of the west coast North American octocorals.

Recently collected material from British Columbia and California has allowed for the examination and taxonomic assessment of several shallow-water soft corals (intertidal to 20 meters in depth), as well as a plexaurid gorgonian (32-85 m).

## Materials and methods

All material examined is housed in the marine invertebrate collections of the California Academy of Sciences, preserved in 95% ethanol, and acquired from various sources. Scanning electron micrographs were made using a LEO 1450 VP SEM.

Material used for comparative purposes: *Euplexaura* sp., CAS 107595, Western Pacific Ocean, Palau, Neco Channel, 28 September 1996, 24 m depth, coll. Gary C. Williams, one whole specimen.

Abbreviation used in the text: **CAS** (California Academy of Sciences, San Francisco).

## Systematic account

### Order Alcyonacea Lamouroux, 1816. Family Clavulariidae Hickson, 1894. Genus *Cryptophyton* Williams, 2000

#### Cryptophyton jedsmithi sp. n.

urn:lsid:zoobank.org:act:482B9A4A-2E2E-4A4A-A319-5CC211242B45

http://species-id.net/wiki/Cryptophyton_jedsmithi

**Figure 1.**
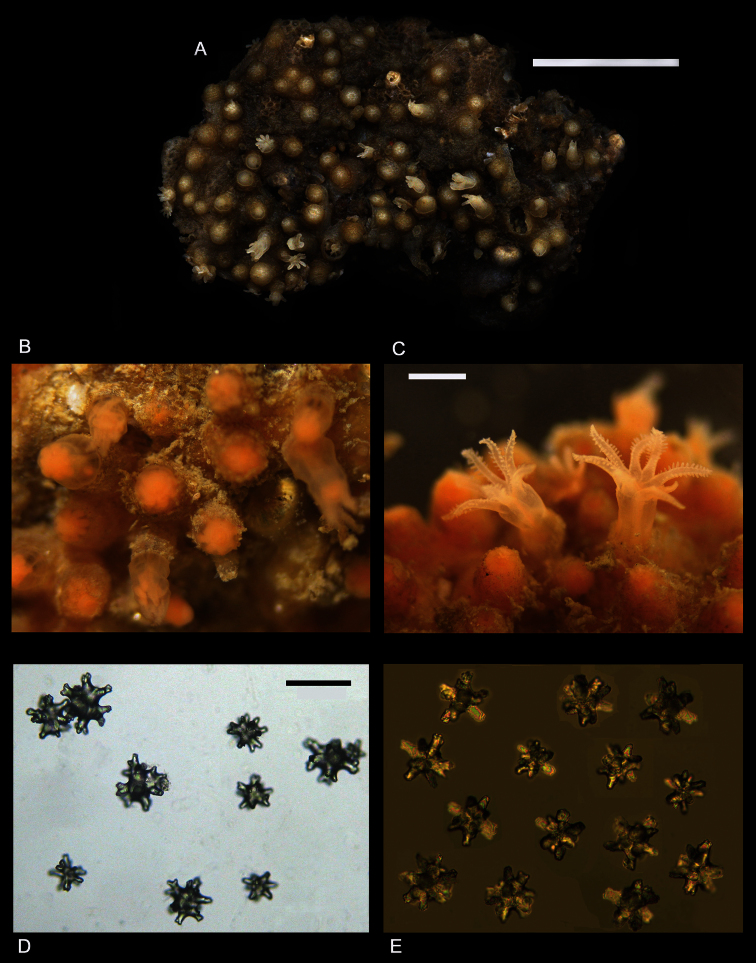
*Cryptophyton jedsmithi* sp. n. **A** Wet preserved holotype (CAS 177194); scale bar = 10 mm. **B–C** Living holotype, details of polyps; photos courtesy of Jeff Goddard; scale bar for both = 1.5 mm. **D–E** Light micrographs of coenenchymal sclerites; scale bar for both = 0.10 mm.

Figures 1 [Fig F2] [Fig F3] –4 , 19

##### Species diagnosis.

Stolons ribbon-like to somewhat broadened in some areas. Anthosteles hemispherical, arise directly from basal stolons, elevated stolonic bars or transverse platforms absent. Anthocodial armature absent. Sclerites of stolons and anthosteles 0.06–0.10 mm in diameter, mostly spiny balls or stellate bodies with projecting processes in three dimensions.

##### Type material.

Holotype. CAS 177194. North America, U.S.A., California, San Diego County, San Diego, Point Loma, 32°42'N, 117°15'20"W, 12 February 2006, collector: Jeff Goddard, one specimen wet-preserved in 95% ethanol.

##### Habitat and distribution

(Figure 19): Under a boulder in the low rocky intertidal zone at the type locality.

##### Etymology.

The species is named for Jedediah Strong Smith, American trailblazer and cartographer, who explored vast regions of western North America between 1822 and 1831, and along the Pacific Coast, including San Diego in December of 1826 (Brooks 1977) – the area of the type locality of the new species.

##### Description.

**Figure 2. F2:**
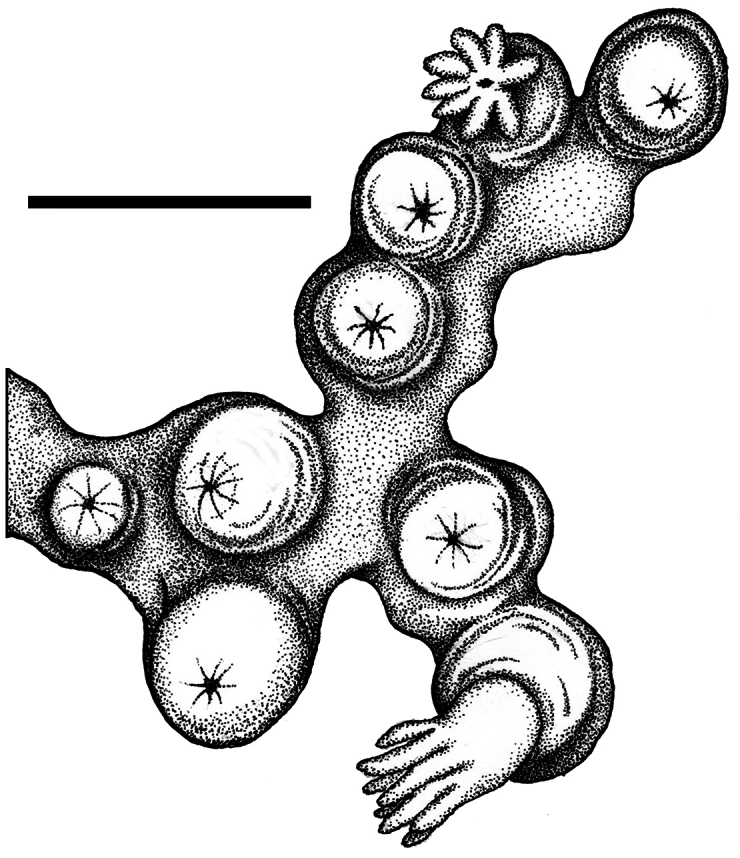
*Cryptophyton jedsmithi* sp. n. A portion of the holotype, showing arrangement of nine polyps on a membranous stolon; scale bar = 3.0 mm.

*Colonial morphology* (Figures 1A, 2). The holotype consists of approximately eighty-five polyps arising from flattened basal stolons. The stolons encrust a piece of dead cheilostomatid bryozoan, 32 mm long by 20 mm wide. The surface of the bryozoan is interspersed with several calcareous tubes of a serpulid polychaete.

*Polyps* (Figures 1B–C). Anthosteles are moundlike, rounded, hemispherical to subcylindrical. Anthocodiae are mostly retracted within the anthosteles, although a few are emergent. The anthosteles are approximately equal in height and diameter, mostly 1–1.4 mm.

**Figure 3. F3:**
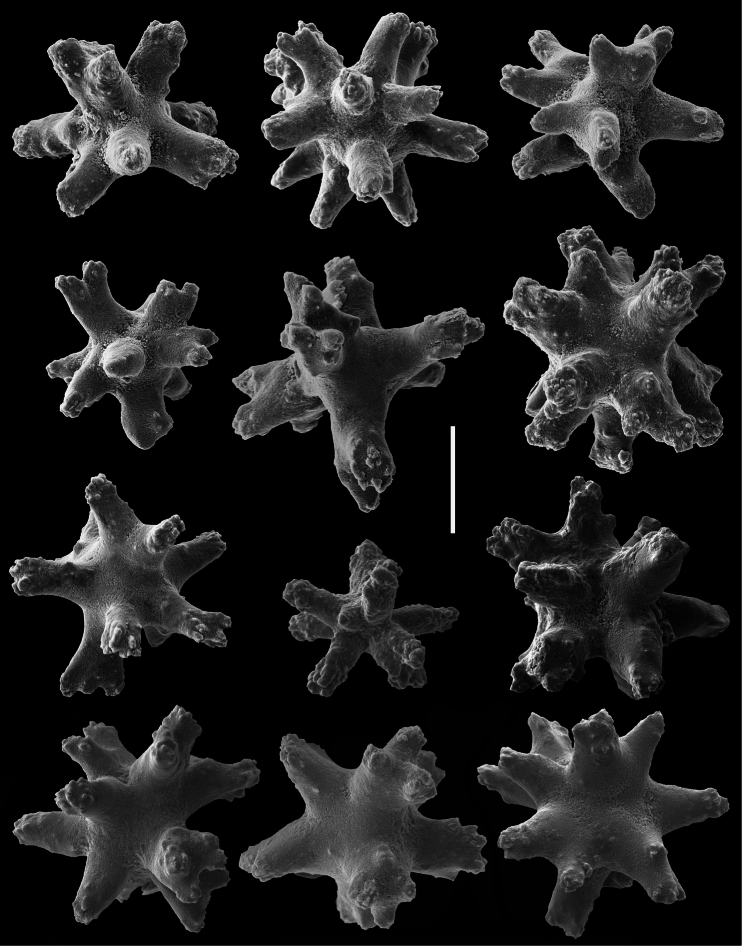
*Cryptophyton jedsmithi* sp. n. Scanning electron micrographs of coenenchymal sclerites from the holotype. Scale bar = 0.03 mm.

**Figure 4.**
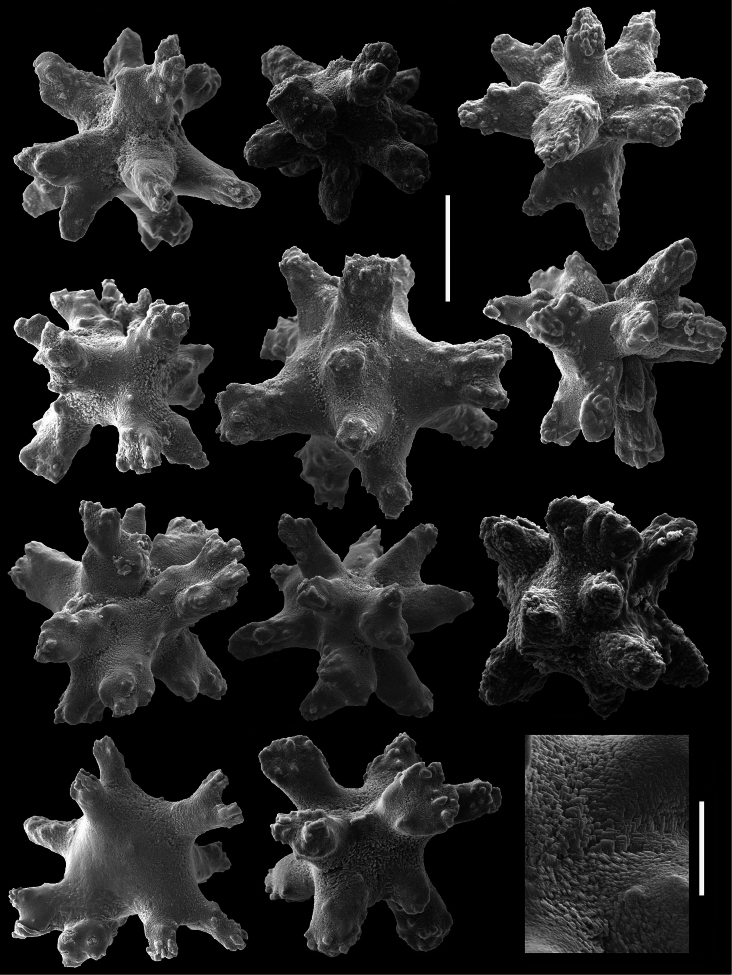
*Cryptophyton jedsmithi* sp. n. Scanning electron micrographs of coenenchymal sclerites from the holotype; scale bar = 0.03 mm. Lower right, ultrastructural detail from center of the sclerite to the adjacent left; scale bar = 0.01mm.

*Sclerites* (Figures 1D–E, 3, 4). Sclerites of the coenencyme and anthosteles resemble spiny balls or stellate bodies with projecting processes in three dimensions; 0.05 – 0.10 mm long. Sclerites are absent from the anthocodiae and polyp bodies.

*Color* (Figures 1A–C). Color in life: the anthosteles are pale orange and the anthocodiae are white. Wet-preserved holotype: stolons and anthosteles light grayish white, while the emergent anthocodiae are white.

##### Differential diagnosis.

Two species of the genus *Cryptophyton* are known, *Cryptophyton goddardi* Williams, 2000 and *Cryptophyton jedsmithi* sp. n. The two species differ in sclerite shape. Those of *Cryptophyton goddardi* are irregularly-shaped radiates, tuberculated rods, and shuttles (Williams 2000: 337), while those of *Cryptophyton jedsmithi* sp. n. mostly resemble spiny balls or stellate bodies (Figures 1D–E, 3–4).

The geographic range of *Cryptophyton goddardi* was originally known only from the type locality of central Oregon on the Pacific Coast of the United States, but has recently been extended southwards to southern California, and has been collected at seven locations (Figure 19), while *Cryptophyton jedsmithi* sp. n. is known only from the type locality – San Diego, California (Figure 19).

##### Key to the species of *Cryptophyton*

**Table:** 

1a	Anthosteles (polyp mounds) cylindrical, up to 2.8 mm in height and 1.8 mm in diameter. Sclerites are irregulary-shaped radiates, irregular sclerites presumably derived from radiates, tuberculate rods, and shuttles; 0.06–0.18 mm long	*Cryptophyton goddardi*
1b	Anthosteles (polyp mounds) hemispherical, approximately equal in height and diameter, mostly 1–1.4 mm. Sclerites resemble spiny balls or stellate bodies with projecting processes in three dimensions; 0.05–0.10 mm long	*Cryptophyton jedsmithi* sp. n.

### Family Alcyoniidae Lamouroux, 1812. Genus *Alcyonium* Linnaeus, 1758

#### Alcyonium sp. indet.

Figures 5 –6

##### Synonomy.

*Alcyonium* sp. Williams (2007: 184-185, 188); Williams and Lundsten (2009: 1078).

##### Material examined.

CAS 179450, Canada, British Columbia, Weynton Passage, Plumper Group of islands, Plumper Island, (50°35.501'N, 126°47.997'W), 20 m depth, 10 November 2009, collector: N. McDaniel, one whole colony. CAS 173217, Canada, British Columbia, Strait of Juan de Fuca, Swordfish Island (48°18'36.4"N, 123°34'58.4"W), 6 m depth, 28 September 2009, collectors: C. Blondeau, T. Hill, R. Van Hall, one whole colony, abundant in underwater tunnel with dynamic surge. CAS 029138, U.S.A., Alaska, Arctic Ocean, near Point Barrow, 44 m depth, 29 July 1951, collector: J. Bohlke on R/V ”Ivik”, two whole colonies.

##### Taxonomic assessment.

*Alcyonium* sp. indet. is known from the west coast of North America from Alaska south to British Columbia and California, and has been referred to as *Gersemia rubiformis*, *Capnella rubiformis*, or *Eunephthya rubiformis* in numerous publications (examples: Madsen 1944; Ofwegen 2012). These binomens are based on the entry *Lobularia rubiformis* by Ehrenberg (1834: 282). All previous references that referred to the original author of the species have noted Ehrenberg (1834) as the original source. However, on page 282 of this work, he notes that Pallas was the original author. This fact apparently has eluded the attention of subsequent authors, both in print and in electronic sources as well. Ehrenberg does not identify the date of Pallas’s description, but it is known that Peter Simon Pallas published works on zoophytes and corals between 1766 and 1798 (Bayer 1981a). Ehrenberg also notes the locality of *Lobularia rubiformis* as *“*Mari septentrionali” (= Northern Sea). Needless-to-say, this is ambiguous and could serve to describe the North Atlantic, the Arctic Ocean, or all northern seas including the North Pacific. The name *Gersemia rubiformis* has been applied to a species of soft coral that is reported to occur in polar to temperate regions of the Arctic Ocean and the northwest Atlantic Ocean from the eastern Canada south to North Carolina (Ofwegen 2012b). It has also been reported from the North Pacific Ocean from Alaska south to California and west to Russia (Williams et al. 1987; Ofwegen 2012b).

**Figure 5.**
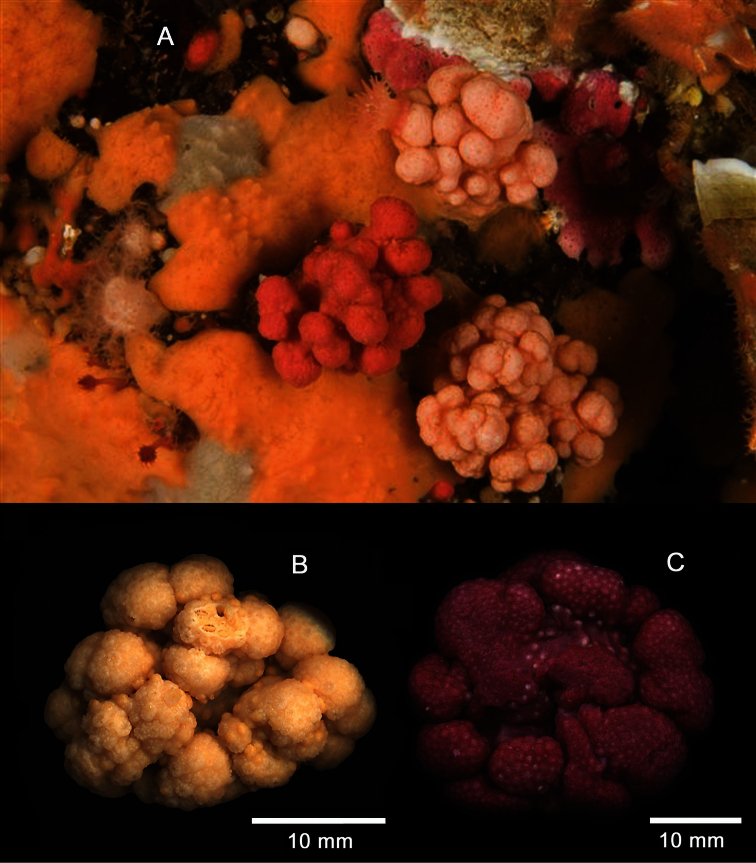
*Alcyonium* sp. indet. **A** Underwater photograph of orange and pale orange colonies, November 10, 2009, at Plumper Rock, Plumper Group of islands, Weynton Passage, British Columbia, Canada, GPS coordinates 50 35.495N × 126 47.998W., 20 m depth. Photo by Neil McDaniel **B** Wet-preserved white specimen (CAS 179450) **C** Wet-preserved red specimen (CAS 173217); scale bars for **B** and **C** =10 mm.

**Figure 6.**
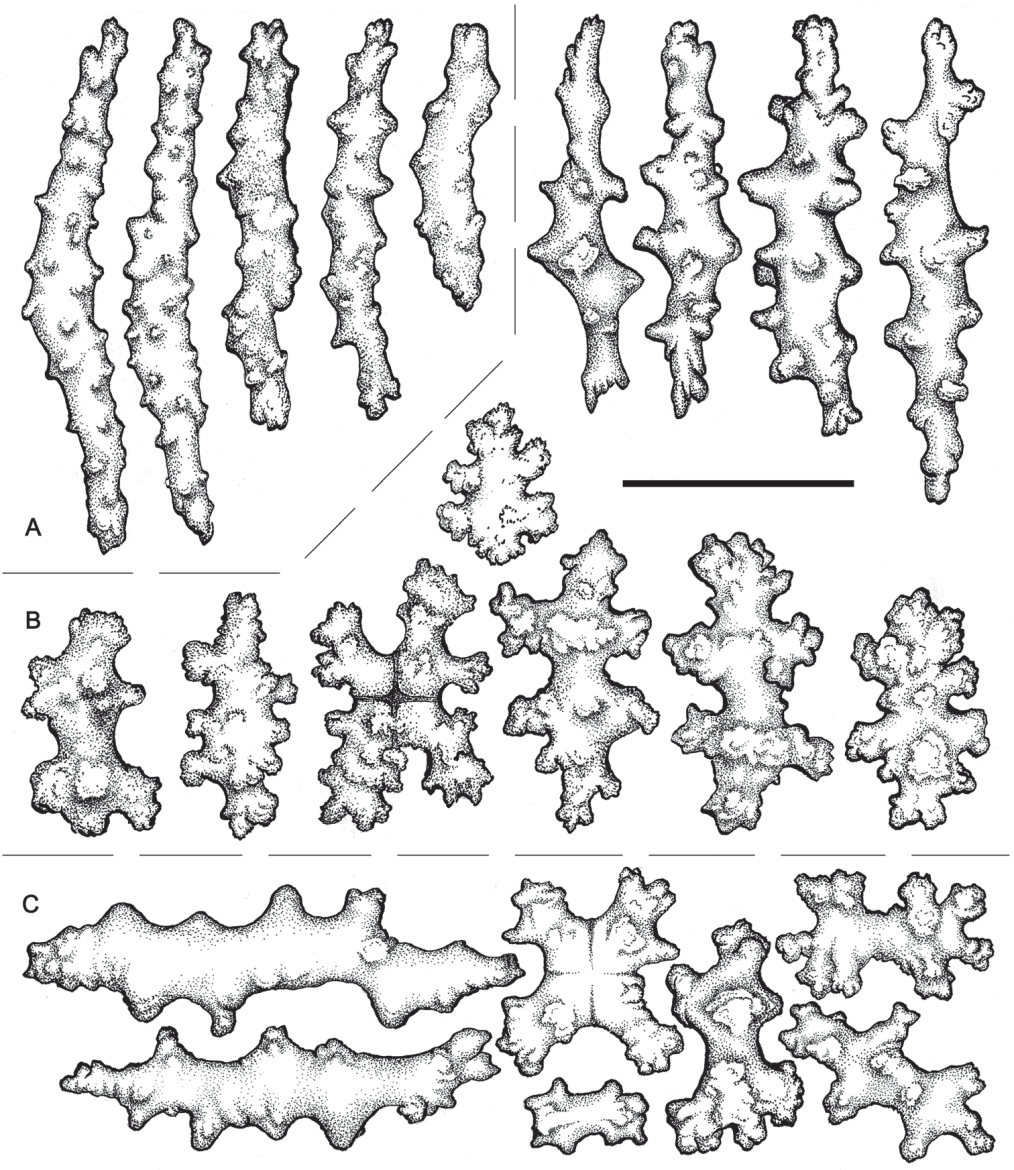
*Alcyonium* sp. indet. (CAS 029138). **A** Polyp sclerites **B** Coenenchymal sclerites of the polypary **C** Coenenchymal sclerites of the stalk. Scale bar = 0.10 mm.

In addition, there are other ambiguous details that are relevant here. Ehrenberg’s 1834 paper is dedicated to the corals of the Red Sea, but he lists *Lobularia rubiformis* as inhabiting the “Mari septentrionali.” The genus *Lobularia* is a synonym of *Cladiella*, a zooxanthellate Indo-Pacific genus that is distributed in the Red Sea as well as much of the Indo-West Pacific (Fabricius and Alderslade 2001). The genera to which this species has been identified in the literature represent two different soft coral families, the Alcyoniidae and the Nephtheidae. My examination of western North American material reveals that the species has small, completely retractile polyps and a polyp arrangement that is lobate rather than catkin-like (even though there may be few to no polyps on the lower parts of the expanded lobes). In addition, the northeastern Pacific material (Figure 5) exhibits morphological similarities to other species of *Alcyonium*, as previously described and illustrated (Verseveldt and Ofwegen 1992; Casas et al. 1997; Ofwegen et al. 2007). The general appearance of the scIerites of *Alcyonium* sp. indet. (Figure 6) are consistent with other species of the genus as well. I therefore here align the species to the Alcyoniidae, rather than the Nephtheidae.

Recent molecular phylogenetic evidence shows that there are two species included in *Gersemia* that nest in the genus *Alcyonium*, rather than with other nephtheids (Breedy et al. 2012: 357). In light of this, future research may show that other species previously allocated to *Gersemia* may in fact belong to *Alcyonium*. It is not known if a type specimen of *Lobularia rubiformis* was ever designated. From the aforementioned, it is here considered that the Pacific coast material cannot justifiably be ascribed to *Gersemia rubiformis* and the validity of that species cannot be determined at present. Because of this, the Pacific coast species is considered as an unidentified species of the genus *Alcyonium* Williams 2007: 184-185, 188; Williams and Lundsten 2009: 1078). It is evident that a taxonomic revision and determination of the validity of *Gersemia rubiformis* is necessary, and that molecular studies of samples from various populations in the Atlantic, Arctic, and Pacific Oceans may provide a clearer understanding regarding taxonomic status.

### Family Nephtheidae Gray, 1862. Genus *Gersemia* Marenzeller, 1878

#### Gersemia lambi sp. n.

urn:lsid:zoobank.org:act:C2BFCF41-3F55-43FA-9858-9FFC2543E0ED

http://species-id.net/wiki/Gersemia_lambi

**Figure 7.**
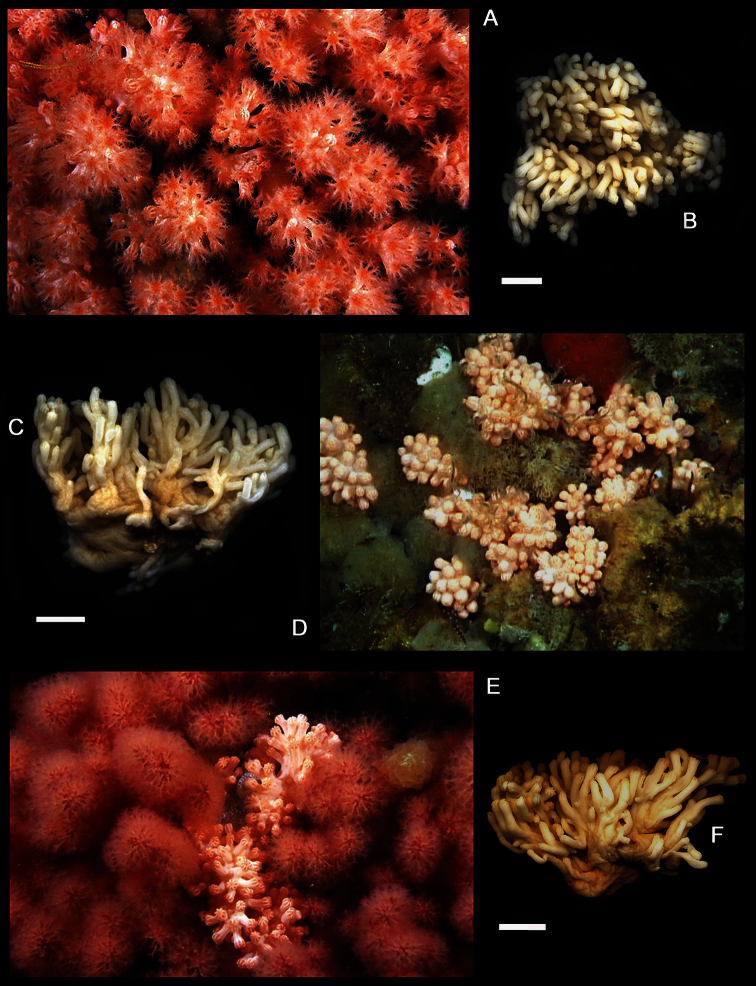
*Gersemia lambi* sp. n. **A** Underwater photograph of colonies with polyps fully expanded; photo courtesy Marc Chamberlain **B** Wet-preserved holotype, dorsal view; scale bar = 10 mm **C** Wet-preserved holotype, lateral view; scale bar = 10 mm **D** Underwater photograph of colonies with polyps retracted; photo courtesy Neil McDaniel **E** Underwater photograph of colonies with polyps retracted (center); photo courtesy Marc Chamberlain **F** Wet-preserved paratype, lateral view (CAS 171941); scale bar = 10 mm.

Figures 7 [Fig F8] [Fig F9] [Fig F10] –11 , 19

##### Species diagnosis.

Polyps clustered in groups on short lobes, emanating from short stalk above holdfast. Polyps tubular, curved, non-retractile, relatively large. Sclerites primarily radiates with variable ornamentation and modification of turberculation; rod-like forms also present. Colonies pink in life, white preserved.

##### Type material.

Holotype. CAS 171939, Canada, British Columbia, Langara Island; 26 June 2004; 12 m depth, collected by Andy Lamb; one specimen. Paratypes. CAS 171940, same data as holotype, one specimen. CAS 171940, same data as holotype, one specimen.

##### Additional material.

CAS 179449, same data as holotype, 11 specimens. CAS 173218. Canada, British Columbia, east side of Kerouard Island, off south end of Queen Charlotte Island; 9 m depth, 51 54.624'N, 130 58.635'N; 7 August 2003; 20 m depth; collected by Doug Swanston; one specimen. CAS 173219. Canada, British Columbia, Queen Charlotte Islands, Kunghit Island, west side of Cape St. James; 21 May 2002; 9 m depth; collected by Danny Kent; one specimen.

##### Habitat and distribution

(Figure 19). Shallow subtidal region from Cape Ommaney, southeast Alaska, USA (according to Neil McDaniel, pers. comm.), to central British Columbia, Canada; 9–20 m depth.

##### Etymology.

This species is named for marine naturalist and educator Andy Lamb (Vancouver, British Columbia), who collected the type material.

##### Description.

*Colonial morphology* (Figures 7B-C). The holotype measures 55 mm in length, 39 mm in width, and 35 mm in height. It is composed of dense concentrations of autozooids distributed in isolated clusters on several lobes that emanate from the stalk, which arises immediately above the basal holdfast. Each cluster usually contains approximately 5 and 15 polyps.

*Polyps* (Figures 7B–C, F). The polyps are monomorphic, non-retractile, tubular in shape, and vary in length and width (4.0–7.0 mm in length and 1.5–2.0 mm in width). The width of the polyps is greatest at the distal extremities. The polyps are erect and often curve upward from their bases.

**Figure 8. F8:**
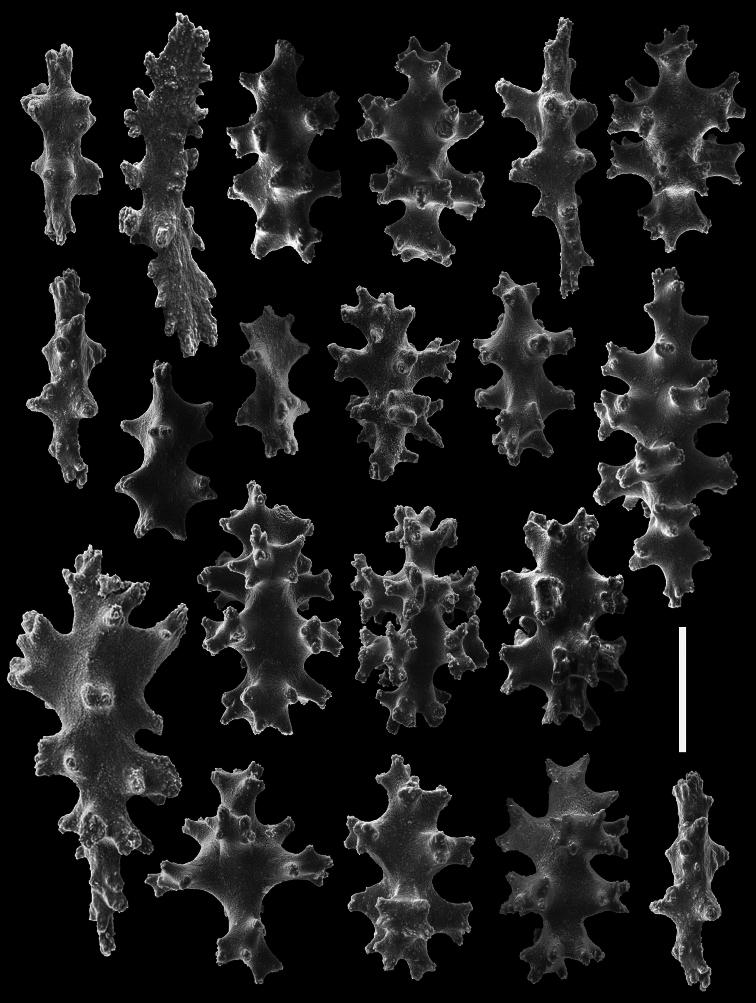
*Gersemia lambi* sp. n. Scanning electron micrographs of polyp sclerites. Scale bar = 0.04 mm.

**Figure 9. F9:**
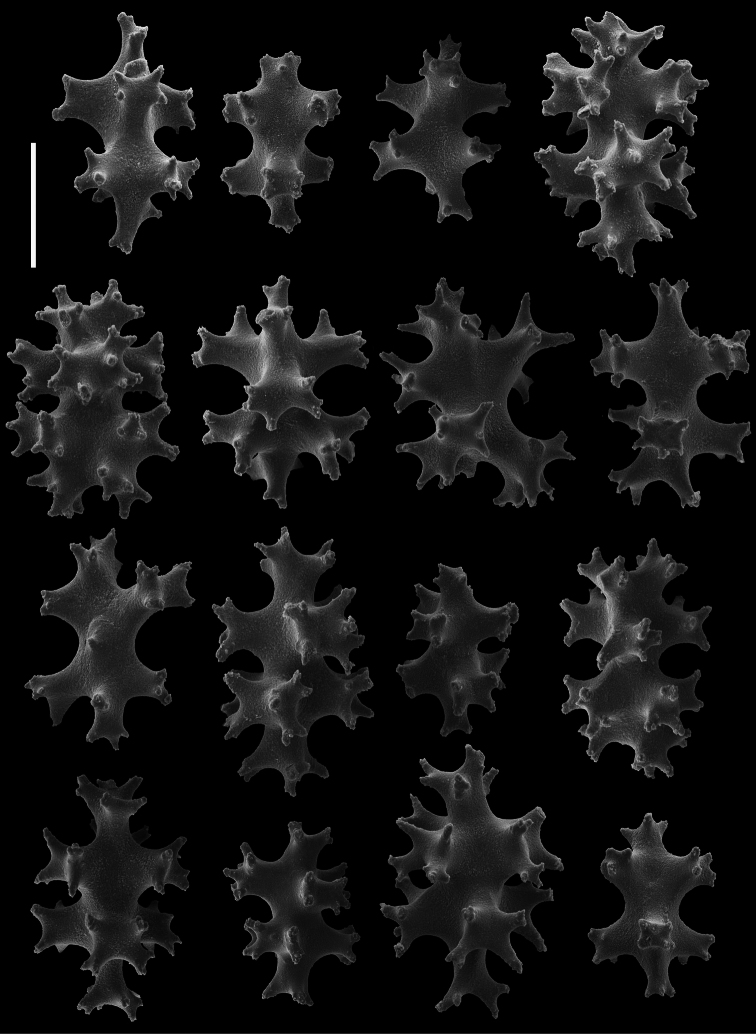
*Gersemia lambi* sp. n. Scanning electron micrographs of sclerites of the polypary coenenchyme. Scale bar = 0.04 mm.

**Figure 10. F10:**
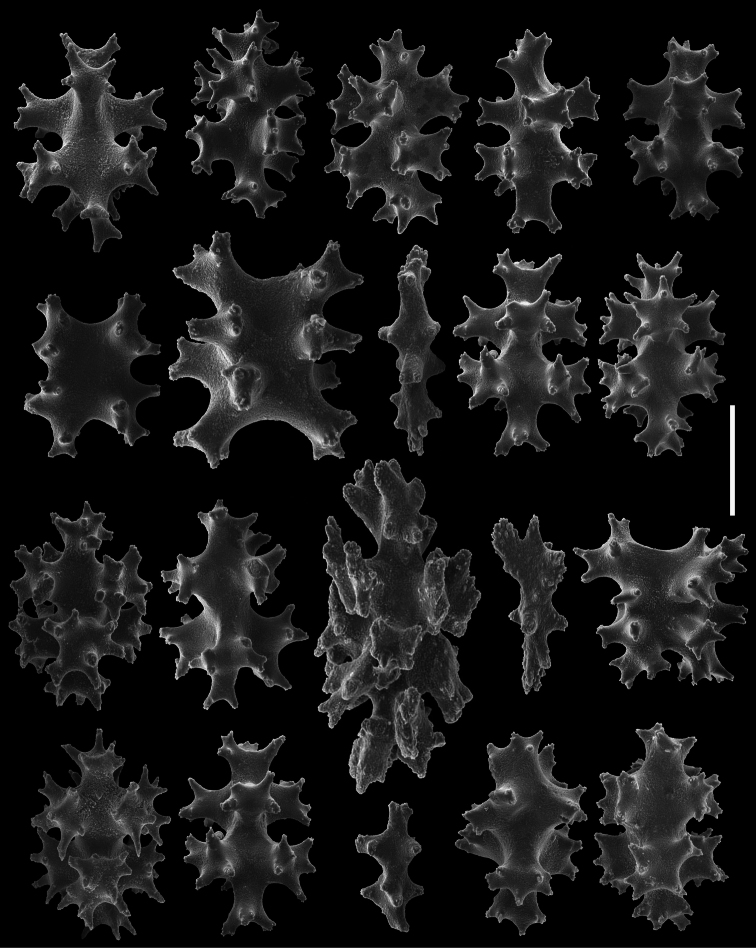
*Gersemia lambi* sp. n. Scanning electron micrographs of stalk sclerites. Scale bar = 0.04 mm.

**Figure 11.**
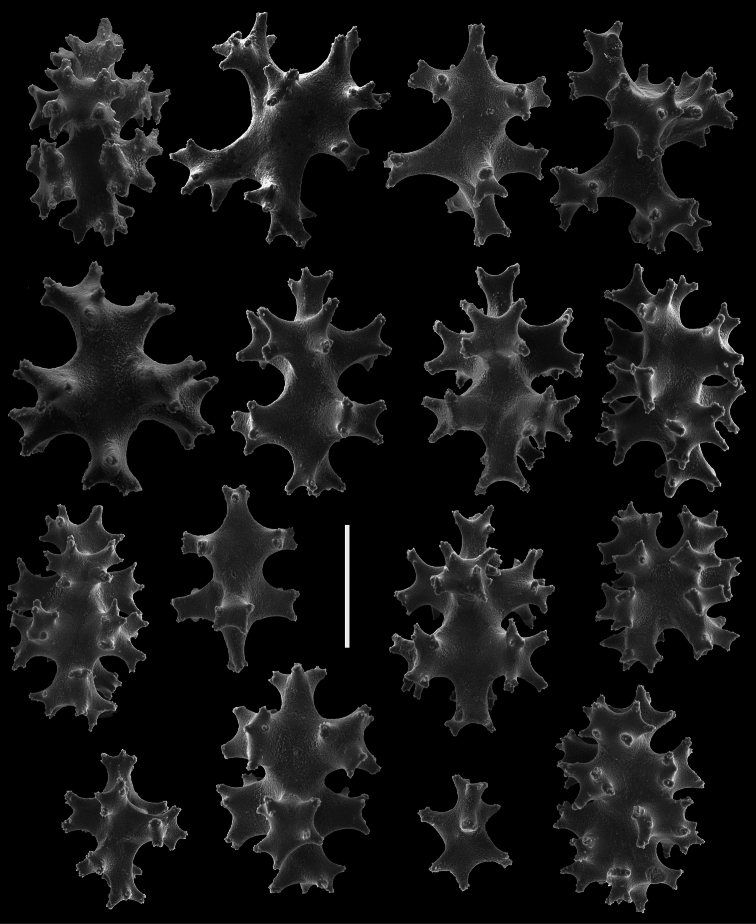
*Gersemia lambi* sp. n. Scanning electron micrographs of stalk sclerites. Scale bar = 0.04 mm.

*Sclerites* (Figures 8–11). Coenenchymal sclerites of the polypary are primarily sharply-tuberculated radiates, 0.03–0.11 mm. Coenenchymal sclerites of the stalk are mostly variously-ornamented radiates and modified radiates, 0.03–0.12 mm long. Polyp wall sclerites abundant, uniformly and densely-distributed, 0.03–013 mm in length, mostly variably-shaped radiates and rods with a few irregularly-shaped elongate forms and crosses. Tentacle sclerites densely and uniformly distributed (not arranged *en chevron*), mostly radiates and rods, although a few club-shaped or approach torch-like forms are also present.

*Color* (Figures 7A–D, E). Colony color is pink to reddish in life, often with orange oral discs. Colonies are uniformly cream-white in color when preserved in ethanol.

##### Differential diagnosis.

The only other species known in the genus *Gersemia* from the Pacific Coast of North America is *Gersemia juliepackardae* Williams & Lundsten, 2009. Although coenenchymal sclerites of the two species are predominantly eight radiates, *Gersemia juliepackardae* and *Gersemia lambi* sp. n. differ markedly in surface feature characters. Those of *Gersemia juliepackardae* are narrow with slender medial waists and relatively rounded tubercle tips (Williams and Lundsten 2009), while those of *Gersemia lambi* sp. n. are broad with wide medial waists and more acute tubercle apexes (Figures 8–11).

This species is distributed from Washington to southern California, while *Gersemia lambi* is known from southern Alaska to British Columbia. The two species differ markedly in their bathymetric distributions. Collected material of *Gersemia juliepackardae* is recorded between 888 to 1600 meters in depth, although video images record the species from 520–2034 meters (Williams and Lundsten 2009). *Gersemia lambi* sp. n., on the other hand, is known at present only from a depth range of 9-20 meters.

**Key to the species of *Gersemia* from the west coast of North America**

**Table:** 

1a	Alcohol-preserved polyps cylindrical, straight, 4.5–5.5 mm long by 1.2–1.5 mm wide. Sclerites of the distal half of polyps are red, all other sclerites colorless. Color of preserved colonies white with pink distal regions of polyps	*Gersemia juliepackardae*
1b	Alcohol-preserved polyps tubular in shape, often curved, 4.5-8.0 mm long by 1.5–2.0 mm wide. All sclerites are colorless. Color of preserved colonies is white throughout	*Gersemia lambi* sp. n.

### Family Plexauridae Gray, 1859

#### Genus Chromoplexaura gen. n.

urn:lsid:zoobank.org:act:72A0D6D2-C439-4B23-875A-1AAEF04B4C2E

http://species-id.net/wiki/Chromoplexaura

**Figure 12.**
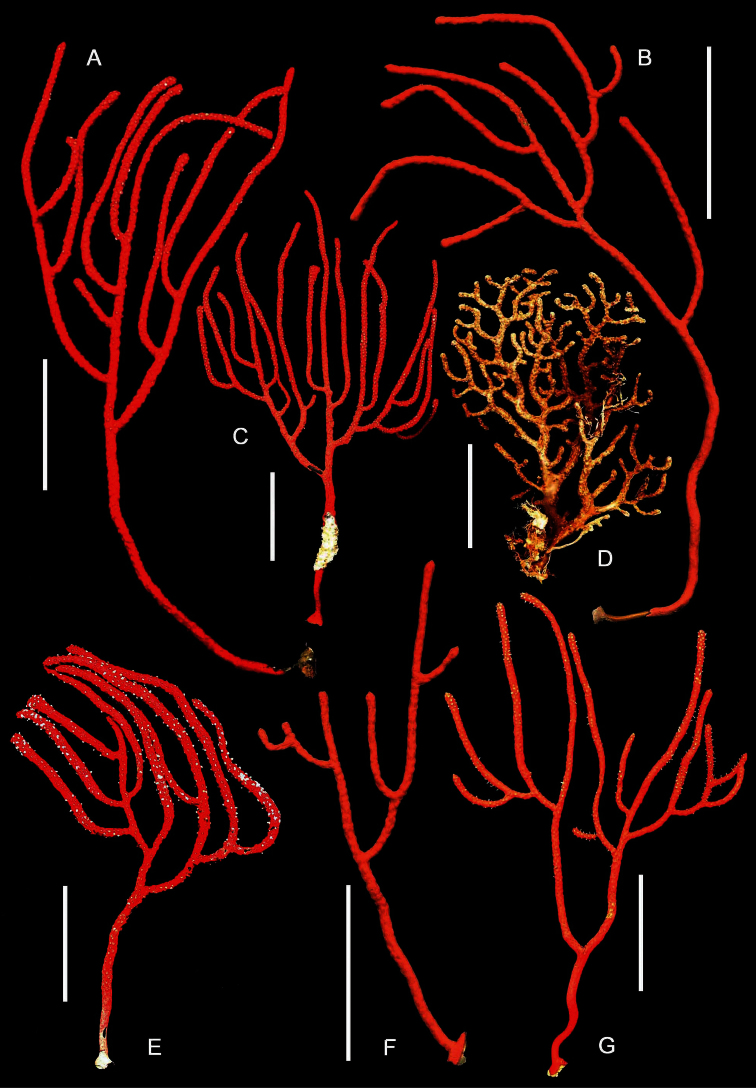
Plexaurid gorgonians, entire colonies. **A, C** *Chromoplexaura marki* (CAS 173222) **B, F, G** *Chromoplexaura marki* (CAS 096746) **D** *Euplexaura* sp. (CAS 107595.) **E** *Chromoplexaura marki* (CAS 168895). Scale bars = 50 mm.

Figures 12 [Fig F13] [Fig F14] [Fig F15] [Fig F16] –17 , 19

##### Generic diagnosis.

Plexaurid gorgonians. Colonies tall, erect, planar. Branching lateral from single basal stem. Upper branches relatively sparse, slender, elongate, mostly slightly curved. Retracted polyps as numerous low rounded protuberances all round surfaces of branches and stem. Sclerites mostly robust spindles and radiates, some ellipsoid to sub-spherical in shape.

##### Type species.

*Euplexaura marki* Kükenthal, 1913.

##### Etymology.

The generic name is derived from the Greek *chroma* (color), and the gorgonian generic name *Plexaura*, in reference to the vivid color of the colonies.

##### Systematics and phylogenetic assessment.

The genus *Euplexaura* (Figures 12D, 18) is an Indo-Pacific genus (eastern Africa to the central Pacific) of 37 named species (Ofwegen 2012a), with the number of valid species not determined. The surface coenenchyme contains numerous robust ovoid to subspherical sclerites (Fabricius and Alderslade 2001). All sclerites are colorless. Since *Euplexaura marki* differs in these several respects to the genus to which is was originally described, a new genus is here named to accommodate it.

A comparison is also warranted between *Chromoplexaura* and two plexaurid genera that share some superficial similarities – *Thesea* with 31 described species from the Atlantic Ocean (Deichmann 1936: 110; Humann and DeLoach 2002: 78; Ofwegen 2012d), and *Swiftia* with 14 described species from the Atlantic and eastern Pacific (Deichmann 1936: 185; Goldberg 2001: 100; Humann and DeLoach 2002: 65; Ofwegen 2012c). It is yet to be determined if the Pacific species presently allocated to *Swiftia* do indeed belong to that genus or another one. The polyps of *Swiftia* emanate from calyx-like protuberances, and both *Thesea* and *Swiftia* have a preponderance of narrow/elongate to robust spindles. The sclerite complements of both genera are composed mostly of spindles with few to no radiates present. On the other hand, the sclerite complement of *Chromoplexaura* is comprised primarily of radiates and variably-shaped spindles.

Forty five genera of the holaxonian family Plexauridae are currently considered valid (Williams and Cairns 2011). Molecular phylogenetic studies have shown that the resolution necessary to produce a molecular phylogeny of the octocorals is lacking at present, due to slow rate of mitochondrial gene changes and a paucity of markers necessary to distinguish some taxa. Regarding future research, it is anticipated that ITS2 sequence data will produce improvements regarding better resolution and more credible results (McFadden et al. 2006, McFadden et al. 2010, Wirshing et al. 2005).

Currently, the evidence based on molecular data does not support the family as being a monophyletic one, but rather has shown many genera dispersed throughout richly-populated phylogenetic trees, and do not exhibit close affinities as a group. In some cases, plexaurid genera have even appeared associated with genera in other families (McFadden et al. 2006; Wirshing et al. 2005). At present, it is therefore not possible to produce a plausible topology of phylogenetic relationships for such genera as *Euplexaura*, *Swiftia*, *Thesea* and *Chromoplexaura* gen. n.

**Figure 13. F13:**
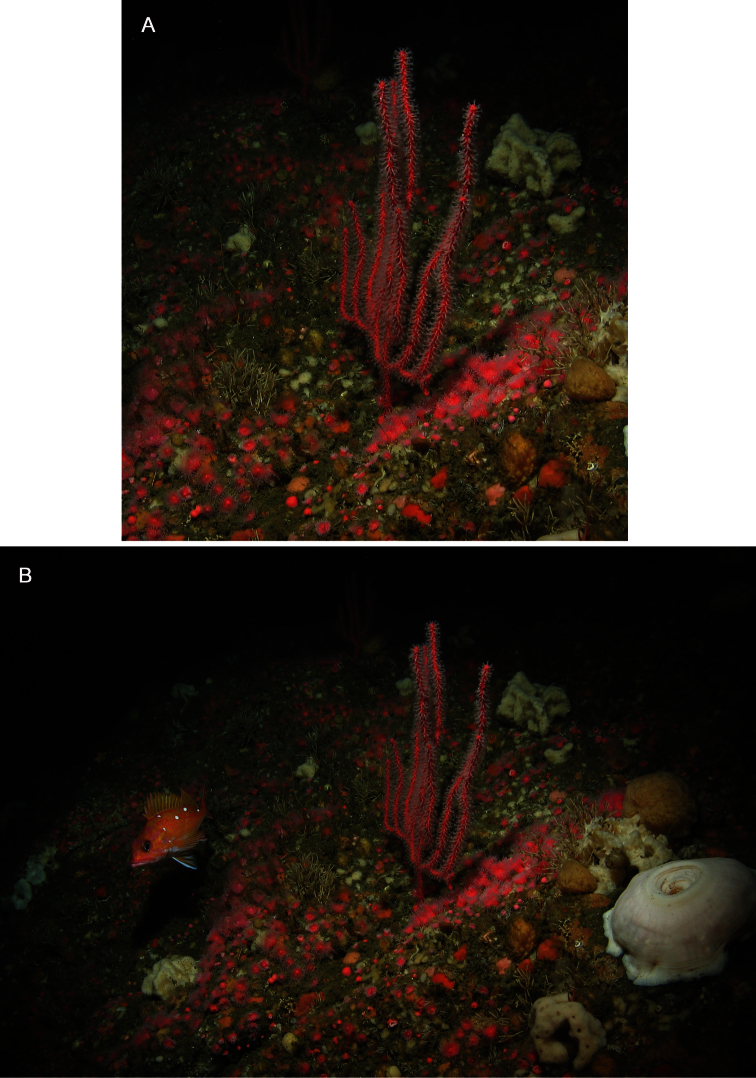
*Chromoplexaura marki*, two living colonies *in situ*. **A** Detail of two gorgonians **B** Wide angle view showing the area inhabited by the colonies. Rittenburg Bank, Gulf of the Farallones National Marine Sanctuary, California, 83 m depth. Photo courtesy: NOAA (National Oceanic and Atmospheric Administration).

**Figure 14. F14:**
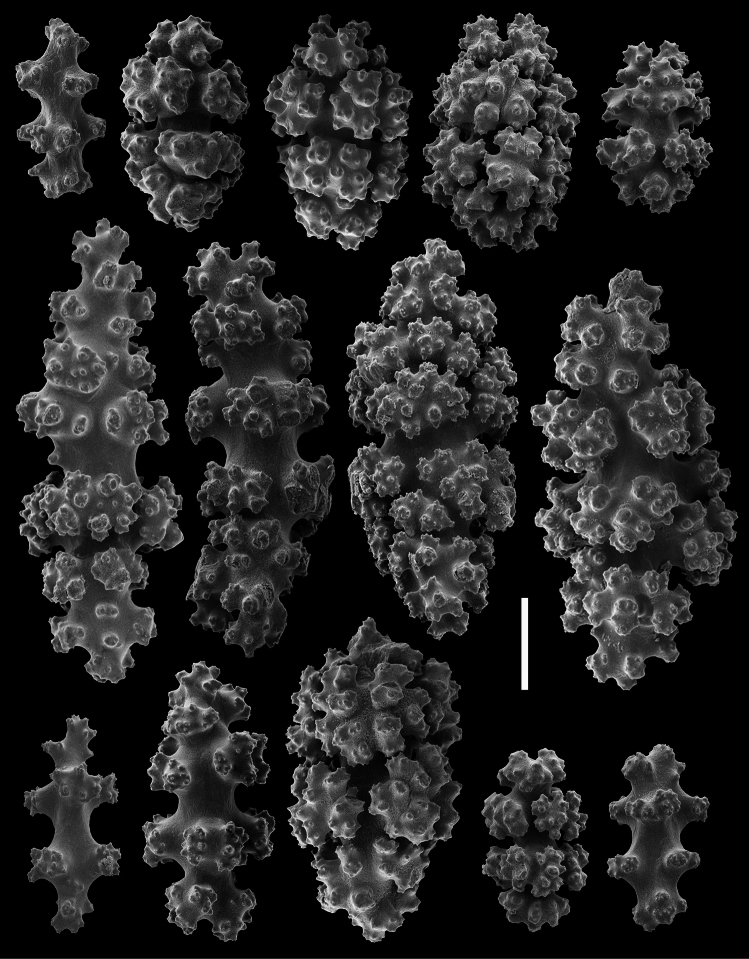
*Chromoplexaura marki* (CAS 096746). Scanning electron micrographs of coenenchymal sclerites. Scale bar = 0.04 mm.

**Figure 15. F15:**
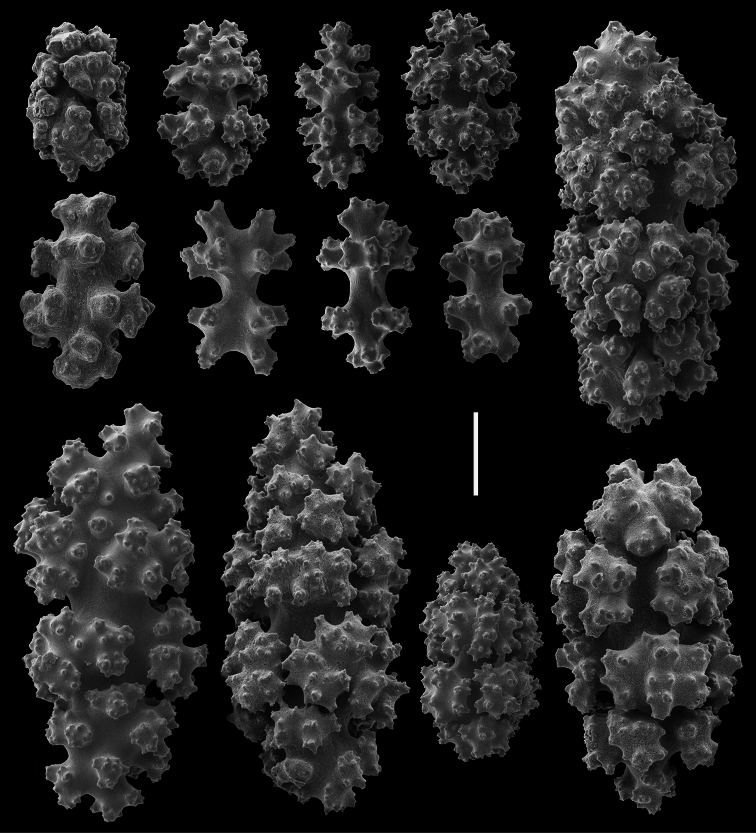
*Chromoplexaura marki* (CAS 096746). Scanning electron micrographs of coenenchymal sclerites. Scale bar = 0.04 mm.

**Figure 16. F16:**
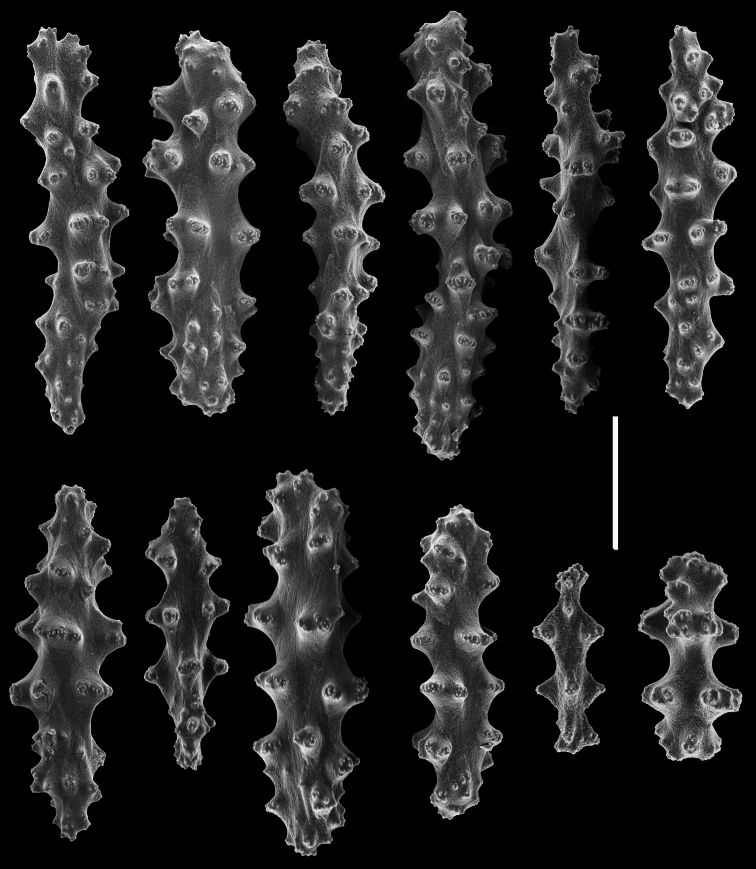
*Chromoplexaura marki* (CAS 168895). Scanning electron micrographs of the polyp sclerites. Scale bar = 0.04 mm.

**Figure 17.**
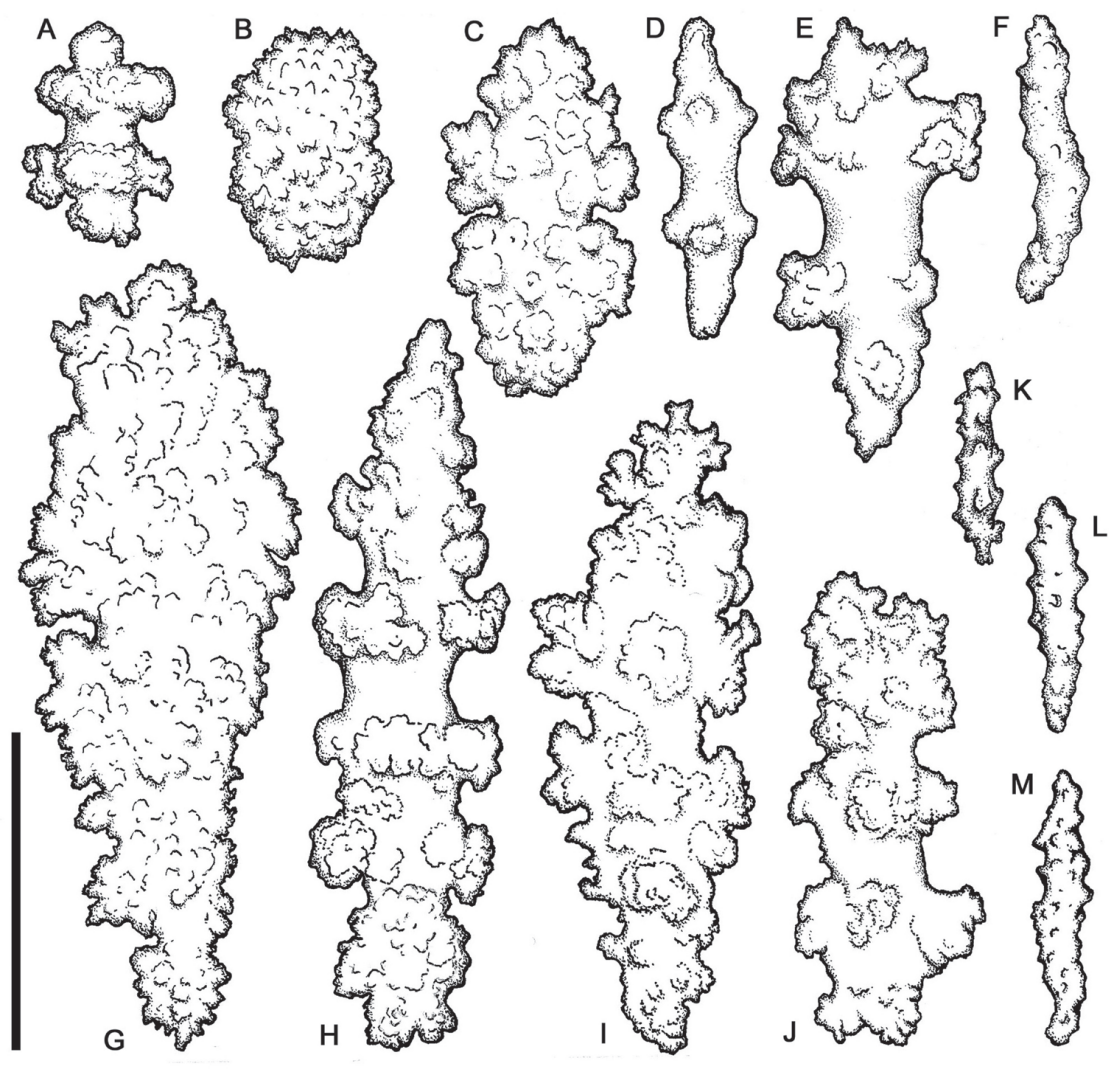
*Chromoplexaura marki*. Variation in sclerite form. **A, B, D, E, F, H, L, M** (CAS 096766) **C, G, I, J, K** (CAS 173222). Scale bar = 0.10 mm.

**Figure 18.**
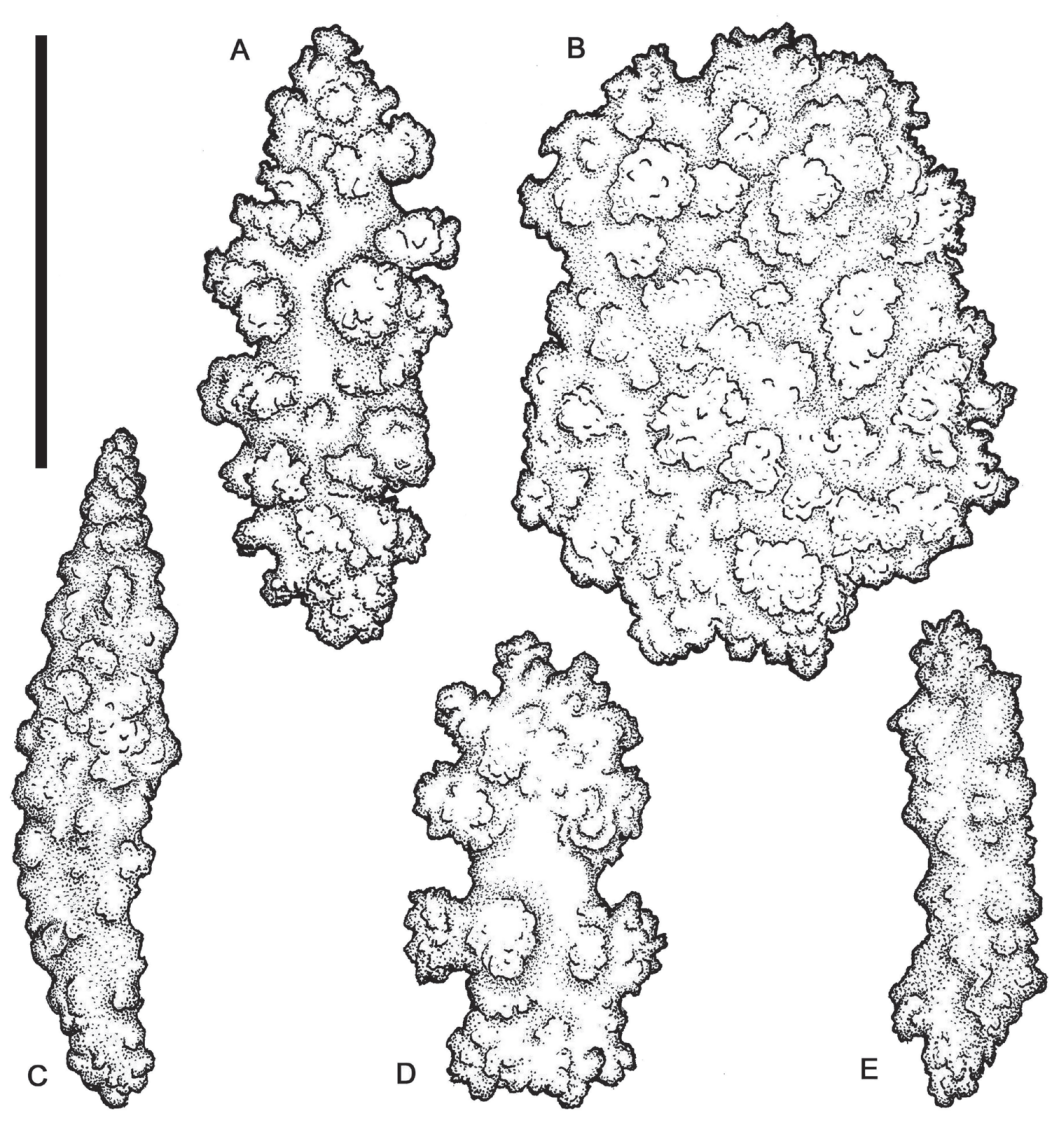
*Euplexaura* sp. (CAS 107595). **A, B, D** Coenenchymal sclerites **C, E** Polyp sclerites. Scale bar = 0.10 mm.

**Key to the genera *Chromoplexaua* and *Euplexaura***

**Table:** 

1a	Colony color red, formed by permanent red coloration of sclerites. Sclerites are mostly radiates and spindles, <0.10–0.25 mm long. One species, temperate Eastern Pacific (California to Oregon)	*Chromoplexaura*
1b	Colony color highly variable, often formed by alcohol-soluble pigments, sclerites colorless. Sclerites are robust ovals to subsperoids and plump spindles, >0.10–0.30 mm long. Approximately 36 described species, tropical Indo-West Pacific (East Africa to Western Pacific)	*Euplexaura*

#### Chromoplexaura marki

(Kükenthal, 1913)

http://species-id.net/wiki/Chromoplexaura_marki

Figures 12 [Fig F13] [Fig F14] [Fig F15] [Fig F16] –17

##### Synonymy.

*Euplexaura marki* Kükenthal 1913: 266; Kükenthal 1924: 93–94; Ofwegen 2012a.

##### Material examined.

CAS 096746, California, Monterey Carmel Bay off San Jose Creek Beach (Monastery Beach), 38 m depth, 20 May 1962, coll. Dennis Sullivan, five whole colonies. CAS 173222, California, Monterey Bay, Carmel Bay (Monterey Bay Marine Sanctuary), 32 m depth, 22 September 2010, coll. Karen Grimmer, two whole colonies. CAS 168895, California, (Gulf of the Farallones National Marine Sanctuary, Rittenburg Bank), 85 m depth, 8 October 2012, coll. National Oceanic and Atmospheric Administration, one whole colony.

##### Description.

*Colonial morphology* (Figures 12–13): The predominantly proteinaceous central axis has a hollow core. The main stem above the holdfast varies from 50–120 mm in length. The ultimate branches measure 10–115 mm in length by 2.5 –4.0 mm wide. The distal extremities are acute to rounded and often slightly swollen compared to the uniform width of the rest of the branches.

*Polyps* (Figures 12–13). Most of the polyps are fully retractile and form low rounded to hemispherical protuberances that are distributed on all sides of the branches. Some polyps are partially exserted and are <1.0 mm in width. Autozooid walls with eight longitudinal rows of densely-set, more-or-less *en chevron* sclerites that give rise to narrow points in the middle of each tentacle.

*Sclerites* (Figures 14–17). The coenenchymal sclerites are radiates, robust spindles, and ovoid forms with highly variable tuberculation, 0.06-0.24 mm in length (Figures 14, 15, 17A–E, G–J). Some are robust and subspherical to ellipsoid with numerous and less well-pronounced tubercles. The sclerites of the polyp wall and points are heavily tuberculated spindles and rods, 0.04 mm–0.09 mm in length (Figures 16; 17F, K, L, M).

*Color* (Figures 12–13). The color of the colonies is similar in life or preserved, the coenenchyme is uniformly- colored orange-red to vivid red, while the exsert polyps are white (Figure 11E). The coenenchymal sclerites are red-orange, while the polyp wall and points sclerites are colorless.

**Figure 19.**
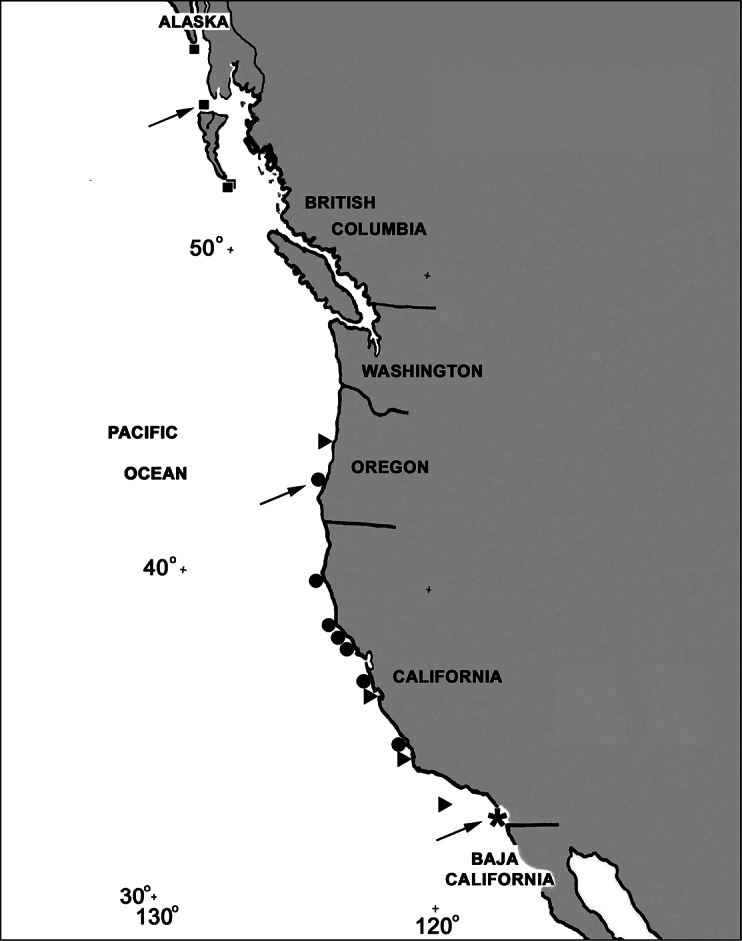
Map of the North American Pacific coast showing collecting localities for *Cryptophyton jedsmithi* sp. n. (*), *Cryptophyton goddardi* (●), *Chromoplexaura marki* (▲), and *Gersemia lambi* sp. n. (■). Arrows designate type localities.

*Distribution* (Figure 19): Central Oregon to southern California; 9 to at least 90 m depth.

*Biology and associated species*. Several of the colonies in lot 096746, have enlargements on the branches that resemble gall-like growths, which contain epizoic barnacles of the genus *Conopea* (pers. comm., R. Van Syoc, California Academy of Sciences).

## Discussion and conclusion

In spite of the fact that the marine fauna of the west coast of the United States is relatively well known with a plethora of marine laboratories dotting the coast, as well as an abundance of well known and excellent manuals and field guides describing the fauna (Morris et al. 1980; Ricketts and Calvin 1985; Niesen 1994; Carlton 2007; Gotshall 2005; Lamb and Hanby 2005), the octocoral fauna is still largely only minimally studied. Perhaps the main factor responsible for this is that although a number of species have been described since the late 19^th^ and early 20^th^ centuries, the essential revisionary systematics necessary for ascertaining valid taxa, has been unfortunately ignored. As an example, the generic designation (*Euplexaura*) for a common plexaurid gorgonian from California and Oregon has been misapplied for the past century (1913–2013). The new genus *Chromoplexaura* is here named to provide a valid designation for the binomen *Chromoplexaura marki*.

In addition, two new species of octocorals are here described from recently collected material in the intertidal zone of southern California and in shallow subtidal regions of British Columbia and southern Alaska.

## Supplementary Material

XML Treatment for Cryptophyton jedsmithi

XML Treatment for Alcyonium

XML Treatment for Gersemia lambi

XML Treatment for Chromoplexaura

XML Treatment for Chromoplexaura marki
